# An Atypical Case of Prolonged COVID-19 Infection

**DOI:** 10.7759/cureus.26921

**Published:** 2022-07-16

**Authors:** Connor Lewis, Akash Gupta, Neil K Gupta

**Affiliations:** 1 Internal Medicine, Yale School of Medicine, New Haven, USA; 2 Internal Medicine, Yale New Haven Hospital, New Haven, USA

**Keywords:** rt pcr, sars cov-2, cycle threshold, immunocompromise, covid-19

## Abstract

Immunocompromised patients with COVID-19 can have prolonged disease courses that require escalation in care to inpatient or ICU settings. We report a case of a prolonged, active COVID-19 infection in an immunocompromised 61-year-old female with a history of non-Hodgkin’s lymphoma. During her hospitalization, her cycle thresholds (CT) continued to worsen despite clinical improvement. We compared our patient’s course and CTs to other reported cases in immunocompromised patients, investigating the efficacy of CTs and their use in evaluating disease progression and severity. RT-PCR tests targeting specific types of replicative viral RNA may have more utility in assessing disease severity and infectivity in immunocompromised patients. Our patient’s disease course, similar to other reported cases, illustrates the need for improved treatment protocols and infection prevention for the immunocompromised population against SARS-CoV-2.

## Introduction

Individuals infected with COVID-19 generally shed the virus from 3-46 days after symptom onset, with asymptomatic individuals shedding comparably to symptomatic individuals in both viral load and duration [1]. However, there have been documented cases of patients remaining RT-PCR positive for COVID-19 for periods of time beyond 46 days [1, 2]. In some of these cases, the patients were in an immunocompromised state with varying severity of disease, ranging from asymptomatic to respiratory failure requiring ICU monitoring [1, 3, 4]. Here, we present a case of an immunocompromised patient who was persistently COVID-positive over a course of nearly two months with close monitoring of her cycle thresholds (CTs). This patient’s course continued to improve despite worsening CT, raising questions about the utility of relying on CT to determine disease severity and recovery in immunocompromised patients.

## Case presentation

Our patient is a 61-year-old female with a past medical history of non-Hodgkin’s lymphoma (NHL), currently in remission. She presented with diffuse muscle weakness and polyarthralgia complicated by limited mobility. Her weakness and pain were so severe that she had been bedridden for the past three days, and she had tested positive for COVID-19 one month prior. She initially presented to her rheumatologist, who recommended she go to the emergency department and be admitted to the hospital. In the emergency department, she was tested and found to be positive for COVID-19 with a CRP of 281.0.

The morning after admission, our patient was febrile to 103^o^F and continued to report muscle and joint pain - especially in the right elbow, wrist, and hand. On exam, she had no rash or signs of synovitis. There was significant tenderness to palpation of both the joints and muscles. A computed tomography scan of the chest taken one week before hospital admission showed patchy, ground glass opacities consistent with COVID-19 pneumonia. A chest X-ray taken during her admission also revealed ground glass opacities and atelectasis. An MRI of the cervical spine without contrast showed no abnormalities. MRI of her right elbow revealed mild intramuscular edema and inflammatory processes. Ultrasounds of her extremities revealed no thrombi. An EMG was performed, which ruled out motor neuropathy, polyradiculopathy, plexopathy, and large fiber polyneuropathy. Our patient’s creatine kinase levels were normal.

On Day 3 of admission, our patient had a ferritin of 1,435 ng/mL and CRP had downtrended to 198.5 mg/L. She continued to test positive for COVID-19 with a CT of 31.8. The patient was started on a course of remdesivir 100 mg IV once a day for five days, dexamethasone 6 mg PO once a day for seven days, and sotrovimab 500 mg IV once to treat persistent COVID-19 in an immunocompromised host. She was also empirically treated with a four-day course of piperacillin/tazobactam for nosocomial pneumonia.

Our patient was thought to have seronegative inflammatory arthritis provoked by her COVID-19 infection. However, treatment of her COVID-19 with remdesivir and dexamethasone did not improve her arthritic symptoms, so rheumatology was consulted. They recommended a five-day course of oral prednisone 20 mg daily. Her right glenohumeral joint was injected with 40 mg of methylprednisolone with minimal improvement. Celecoxib and famotidine were added to her arthritic medication regimen. The patient showed gradual improvement in right shoulder mobility, though it was unclear if the improvement was secondary to any of the interventions. Due to her improvement, she was retested for COVID-19 on Day 20 of admission. Her COVID-19 test remained positive and her CT was steady at 31.9.

On Day 21 of admission, our patient developed a fever of 100.4oF and a drop in blood pressure to the 90s/50s mmHg with a lactate of 4.1 mmol/L, concerning for sepsis. She was put on 2L of oxygen, given 4L of IV fluid, and treated empirically with a course of vancomycin and piperacillin/ tazobactam. A chest computed tomography scan revealed new multifocal opacities and trace pleural and pericardial effusions. These effusions raised concern for possible multisystem inflammatory syndrome in adults (MIS-A), for which a workup was started. During this episode, the patient reported significantly increased, severe pain from her arthritis - especially in her legs bilaterally. Another course of oral prednisone was recommended at 40 mg once a day, as well as a cessation of celecoxib. 

On Day 27 of admission, the patient experienced acute hypoxemic respiratory failure with oxygen saturation decreasing to 54% on 2L nasal cannula, diffuse airspace opacities on chest X-ray, significant ground glass opacities on the chest computed tomography scan, and a BNP of 1588, necessitating transfer to the MICU. There, she was started on a high-flow nasal cannula, transitioned to BiPAP, and eventually intubated. In the MICU, our patient was found to have enterococcus and staph epidermis bacteremia and was treated with a 10-day course of piperacillin/tazobactam. A repeat chest computed tomography scan at this time showed worsening bilateral ground glass opacities. Our patient was still testing positive for COVID-19, with a CT that had worsened to 21.8. She was treated with an additional course of baricitinib and remdesivir. Her status improved with these treatments and, after six days, she was extubated. Despite her clinical improvements, a COVID-19 test on Day 36 was positive with a continually worsening CT of 17.6.

On Day 36, our patient was transferred to the medical floor. After reviewing our patient’s heavy steroid regimen and significant polyarthritis and proximal weakness, the medical team became concerned regarding steroid-induced myopathy. She was started on a long-term steroid taper, decreasing her prednisone dose by 5 mg every three days. She was also started on a 10-day course of piperacillin/tazobactam for enterococcus bacteremia of unclear origins. While on the floor, our patient’s white blood cells continued to trend downward, but she also developed a new oxygen requirement prompting a CTA of her chest. This scan was negative for pulmonary embolism and revealed improving bilateral consolidations and ground glass opacities. However, her oxygen needs continued to escalate, ultimately requiring a non-rebreather mask and a second transfer to the MICU on Day 42. She was also tested for COVID on Day 42, with a positive result and CT of 19.3. 

While in the MICU, she was treated with a high-flow nasal cannula and started on empiric ceftriaxone and doxycycline. She was re-treated for suspected COVID-19 pneumonia with tocilizumab and another course of remdesivir. She was also started on 50 mg of methylprednisolone IV once a day. After a five-day MICU course, the patient was able to be weaned to a nasal cannula (NC) and, on day 46 was transferred to the step-down unit (SDU) for continued monitoring.

During her five-day stay in the SDU, she began to clinically improve. She was successfully weaned off the NC and her arthritic pain gradually improved, though she was still markedly weak. A chest X-ray taken on Day 4 of her stay showed improving bilateral lower-lobe opacities. However, on Day 50 she continued to test positive for COVID-19 at a low CT of 17.1 and was treated with a 175 mg injection of bebtelovimab.

**Figure 1:**
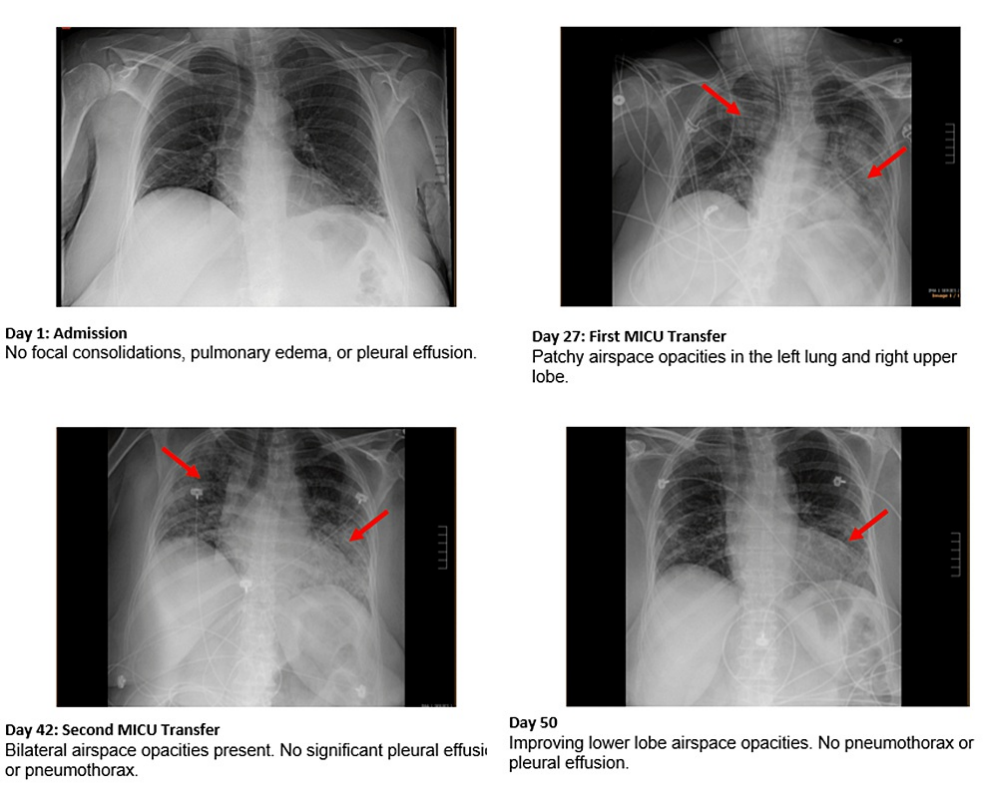
Chest X-rays with summarized radiology reads. These chest X-rays illustrate initial worsening pulmonary disease with eventual improvement in opacities. These improvements occurred despite the worsening of the patient's CT.

Given her clinical and radiographic improvements, she was transferred to the floor on Day 51. Her chest X-rays, demonstrating improvement in lobar consolidations, are illustrated in Figure 1. She was further stabilized and transferred to a long-term rehabilitation facility on Day 55.

## Discussion

**Figure 2:**
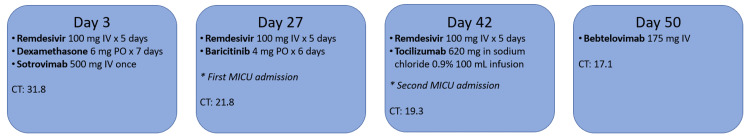
Multiple courses of COVID-19 treatment with corresponding CT at treatment initiation. CT: cycle threshold

Our patient underwent multiple courses of treatment for her COVID infection involving numerous medications and monoclonal antibodies. Initially, she was treated with remdesivir, dexamethasone, and sotrovimab. Methylprednisolone and high doses of prednisone were given to help modulate her immune response and alleviate her arthritic symptoms. Baricitinib was given for both her arthritic symptoms and COVID-19 infection. Later in her disease course, she was given the monoclonal antibody bebtelovimab due to persistently low CTs despite clinical improvement. Figure 2 describes the multiple courses of treatment in greater detail.

Our patient’s active COVID infection of 55 days was significantly longer than what would be expected in an immunocompetent patient but similar in length to other reported cases of COVID-19 infection in immunocompromised patients.

Alsaud et al. reported the case of a 65-year-old male with chronic myeloid leukemia (CML) on dasatinib. He was febrile with bilateral rhonchi and lower lobe consolidations on a chest X-ray. After being diagnosed with a positive COVID-19 RT-PCR test result, the patient experienced a prolonged course of COVID infection with deterioration requiring a seven-day ICU stay. The total duration of his symptoms from the initial test was 35 days, with full radiologic recovery after 47 days. However, this patient continued to have positive RT-PCR test results for a total of 54 days from the initial positive test [3].

Avanzato et al. reported a case of a prolonged COVID infection in a 71-year-old female with chronic lymphocytic leukemia (CLL) and acquired hypogammaglobulinemia. This patient tested positive for COVID-19 with RT-PCR after staying in a rehabilitation facility where a COVID outbreak occurred, although the patient was asymptomatic. She was treated twice with convalescent plasma and received negative RT-PCR test results one month after the second course of treatment. She had positive RT-PCR test results for 105 days after the initial positive test. Her highest viral load was detected on day 70 with a CT of 22.44 [1].

Nakajima et al. reported a case of prolonged COVID in a 47-year-old male with a history of follicular lymphoma in complete remission following six courses of treatment with obinutuzumab plus bendamustine. The patient had been on a bi-monthly maintenance dose of obinutuzumab prior to his positive COVID RT-PCR test result. He was hospitalized in an airborne-isolation ward on day 31 of his infection, had positive RT-PCR test results for 65 days after the initial positive test, and was discharged from the hospital on day 69 [4].

Our case, like previously reported cases of COVID-19 infection in immunocompromised patients, illustrates that prolonged courses of the disease for two to three months are not uncommon for this patient population. These cases may require more intensive management and longer hospitalization than infection in the immunocompetent population.

**Figure 3:**
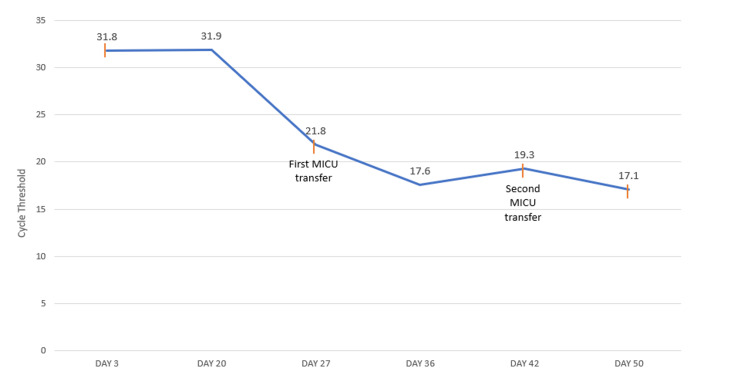
Graph of CT over the patient's hospital course. The orange lines indicate the initiation of a new course of COVID-19 treatment. Specifics of each course of treatment are detailed in Figure 1. CT: cycle threshold

Our patient demonstrated clinical and radiographic improvement despite worsening CT. The persistently worsening CT values are illustrated in Figure 3. This was unexpected since clinical improvement has been associated with increasing CTs. 

A case report by Asai et al. reported 10 cases of COVID-19 infection in immunocompetent patients where CTs were recorded on the initial nasopharyngeal RT-PCR test, as well as subsequent tests throughout the disease course. Of the nine out of ten patients who experienced recovery, all had gradually increasing CT values that mirrored clinical improvement. However, RT-PCR tests remained positive for many of the cases even after symptomatic recovery. A positive RT-PCR test was defined by a CT of <45.0. The clinicians also reported that the live virus could not be cultured after Day 8, despite continued positive RT-PCR results, indicating decreased infectivity at this point [5].

Similarly, a German study found that the live virus could not be cultured after Day 8. These researchers believed that live viral culture was a better indicator of infectivity than an RT-PCR test due to measured levels of subgenomic RNA (sgRNA) [6]. SgRNA is a form of RNA only present in infected cells where viral replication is actively occurring. Additionally, sgRNA does not persist in the absence of viral replication [1]. In this study, throat sgRNA levels were undetectable by Day 5, and sputum sgRNA levels declined from Days 10-11, indicating a cessation of active viral replication. However, these samples continued to have a high viral load and test positive on RT-PCR [6].

A French study found that samples with a CT of 13-17 could culture the live virus. The rate of successful viral culture declined with increasing CT values, with no successful cultures with a CT of greater than 34 [7].

Taken together, these reports indicate that traditional RT-PCR, which may measure numerous types of viral RNA in a given sample, lacks the specificity necessary to determine the degree of active infection. A better indicator of infectivity is the level of sgRNA specifically. This measurement is reflected in the ability to culture the live virus. Additionally, patients may exhibit clinical improvement and even recovery without completely clearing viral RNA from the oropharynx, nasopharynx, or lungs. Additional studies are needed to investigate the degree of infectivity in patients with declining sgRNA levels, and whether sgRNA levels and live viral culture can be used as a benchmark for patients in de-isolation and discharge settings. Ultimately, this could lead to shorter hospitalizations and decreased isolation periods, resulting in a lower overall burden on the healthcare system.

Our case illustrates the need for better prevention of COVID infections in the immunocompromised population. These individuals are at a greater risk of COVID infection because of their decreased immune response to the vaccine as well as their higher likelihood of a prolonged disease course with greater complications.

COVID-19 vaccination in immunocompetent populations has been shown to induce both humoral and cell-mediated immunity. This response involves antibody production against the SARS-CoV-2 spike protein, B memory cells that will continue to produce antibodies, and increased immune activation by CD4+ type 1 helper T cells and CD8+ T cells [8]. In immunocompromised patients, this response is significantly decreased. A study of kidney transplant patients found that only 25% had detectable anti-spike antibodies two to six weeks after completing the two-shot mRNA vaccination series [9]. A study of 500 cancer patients reported that only 66% of patients with hematologic malignancies had detectable levels of anti-spike antibodies [10].

The significantly impaired vaccine response in immunocompromised patients puts these individuals at a much higher risk of contracting a COVID-19 infection. Once infected, these patients may experience prolonged hospitalizations and significant complications, as reported in our case as well as others above.

Studies should be conducted on more effective immunization methods for this vulnerable population, as well as how to mitigate the risk of complicated and prolonged hospital stays. Using measurements of sgRNA and live viral cultures in this population may result in more accurate measures of infectivity.

## Conclusions

This report documents a case of prolonged, active COVID-19 in an immunocompromised female with a history of non-Hodgkin’s lymphoma. Similar to other published reports of COVID-19 in immunocompromised patients, our patient’s disease course lasted for over 50 days and encompassed numerous secondary infections and escalations of care to the ICU level. Due to the risk of prolonged and severe COVID-19 infections in immunocompromised patients, greater infection prevention and treatment measures should be established for this vulnerable population. Our patient’s case is unique because her CT values continued to decline despite clinical and radiographic improvement. CTs may not be an accurate measure of viral infectivity or severity since traditional RT-PCR does not specifically measure sgRNA, which is a better indicator of active viral replication than other forms of viral RNA. Live viral cultures may reflect sgRNA levels and could also be an alternative to traditional RT-PCR. Utilizing these more targeted testing techniques may allow for a greater understanding of disease course and current infectivity.
